# New Hybrid Adsorbents Based on Polyaniline and Polypyrrole with Silicon Dioxide: Synthesis, Characterization, Kinetics, Equilibrium, and Thermodynamic Studies for the Removal of 2,4-Dichlorophenol

**DOI:** 10.3390/polym15092032

**Published:** 2023-04-25

**Authors:** Amina Bekhoukh, Mohamed Kiari, Imane Moulefera, Lilia Sabantina, Abdelghani Benyoucef

**Affiliations:** 1 Department of Process Engineering, Faculty of Science and Technology, University of Mustapha Stambouli Mascara, Mascara 29000, Algeria; 2 Department of Chemical and Physical Sciences, Materials Institute, University of Alicante (UA), 03080 Alicante, Spain; 3 Chemical Engineering Departement, Faculty of Chemistry, Regional Campus of International Excellence “Campus Mare Nostrum”, University of Murcia, 300071 Murcia, Spain; imane.moulefera@um.es; 4 Berlin School of Culture + Design, Berlin University of Applied Sciences-HTW Berlin, 12459 Berlin, Germany; 5 LSTE Laboratory, University of Mustapha Stambouli, Mascara 29000, Algeria

**Keywords:** polypyrrole, polyaniline, 2,4-dichlorophenol, silicon dioxide, adsorption

## Abstract

In the current study, polyaniline and polypyrrole with silicon dioxide (PAni:PPy@SiO_2_) were combined to formulate a new adsorbent, which was examined using XRD, TEM, SEM, FTIR, TGA, and BET, and the adsorption kinetics were investigated by UV–vis spectroscopy. The optical band gap was also evaluated. The electrochemical behavior was investigated using cyclic voltammograms. Moreover, experimental conditions were used to evaluate the 2,4-dichlorophenol (2,4-DCP) adsorption based on the pH, temperature, reaction time, and initial concentration. The analytical isotherm data were determined by Langmuir, Freundlich, Temkin, Sips, and Redlich–Peterson models. For the analysis of the kinetic data, the pseudo-first- and -second-order models and the intraparticle diffusion model were investigated. It was found that this new adsorbent possessed the highest adsorption efficiency after several regeneration cycles. Furthermore, the thermodynamic parameters of adsorption, such as entropy (Δ*S*), enthalpy (Δ*H*), and standard Gibbs were measured. These results suggest that the PAni:PPy backbone can generally be better applied for the elimination of 2,4-dichlorophenol by appropriately dispersing it over the surface of suitable SiO_2_. This search provides a novel way to develop separable, high-performance adsorbents for adsorbing organic contamination from wastewater.

## 1. Introduction

Today, water contamination is a major problem worldwide. Chlorophenols are common organic pollutants used widely in the preparation of many pharmaceuticals and pesticides. Chlorinated phenol waste discharge into water exhibits toxicity to humans, aquatic organisms, and the environment [1,2]. Consequently, 2,4-dichlorophenol (2,4-DCP) is highly toxic, carcinogenic, and mutagenic and is listed as one of the environmental priority pollutants [3,4]. Hence, it is very significant to find a way to eliminate 2,4-DCP. Among the existing treatment methods, including photocatalytic degradation [5], electrochemical oxidation [6], membrane filtration [7], flocculation coagulation [8], adsorption [9], and so on, the adsorption process is one of the more efficient and promising technologies owing to its low cost, simplicity in design and operation, and superior adsorption effectiveness even at low concentrations [9,10,11]. In addition to its toxic effect, the presence of 2,4-DCP in water in even trace amounts (5–10 μg/L) produces a specific odor and makes it undrinkable [1,2,3,4].

Intensive work and the use of conducting polymers (CPs), such as polypyrrole (PPy) and polyaniline (PAni), which are insulators in their neutral state, have been widely promoted in recent years [12,13,14]. CPs serve as redox mediators for electrons and protons between the substrate and electroactive components in the medium. In addition, CPs further improve the interfacial properties between the developed material and the electrolyte, thus facilitating charge transfer from the substrate to the inorganic compound embedded in the CPs [15,16]. Furthermore, both PPy and PAni have π-conjugation in their polymeric structure. Moreover, CPs are generally utilized as matrices to insert inorganic compounds. CP nanocomposites are useful for various medical and environmental applications. They are used in sensors, tissue regeneration, and the remediation of polluted environments [2,4]. Importantly, amorphous SiO_2_ has a porous structure, adsorption properties, and high surface reactivity, which allows it to be used for antimicrobial agents, ceramics, abrasives, adsorption materials, and chemicals [9]. This makes it an excellent candidate for hybrid material applications. Recently, the ternary composites of PAni@ZnO-SiO_2_, PAni@TiO_2_-CuO, and PAni@TiO_2_-TiC were synthesized by our group [2,17,18]. The composites were proposed to incorporate the advantageous properties of the individual compounds. Fortunately, the composites have shown exceptionally improved thermal stability, higher BET surface area, and good electroactivity. Interestingly, the ternary materials have shown better textural properties compared to the binary materials and the separate compounds.

In this context, in the adsorption technique, new adsorbents have been tested to help in the control of pollutants. From this point of view, in this study, a ternary composite of PAni, PPy, and SiO_2_ (PAni:PPy@SiO_2_) was prepared by the in situ oxidative polymerization to be used as a low-cost adsorbent for the removal of 2,4-DCP in aqueous solution. The SiO_2_ penetration into the PAni:PPy network resulted in the generation of porosity in the polymer chains. The porosity and penetration of SiO_2_ lead to good contact between the constituents. Moreover, the prepared adsorbents were characterized by thermogravimetric analysis XRD, UV–vis, TEM, FTIR, TGA, and BET before their application to the adsorption. Their electrochemical behavior was also investigated by analyzing cyclic voltammograms.

## 2. Materials and Method

### 2.1. Materials

Pyrrole (C_4_H_5_N; Sigma Aldrich, St. Louis, MO, USA, 98%), aniline (C_6_H_5_NH_2_; Sigma Aldrich, ≥99.5%), amorphous silicon dioxide (SiO_2_; Sigma Aldrich, 99.5%), ammonia solution (NH_4_OH; Merck, 25%), ammonium persulfate (APS; Merck, Rahway, NJ, USA, ≥98%), N-methylpyrrolidone (NMP; Merck), hydrochloric acid (HCl; Merck, 75%), sodium hydroxide (NaOH; Merck, 37%), 2,4-dichlorophenol (2,4-DCP; Merck, 98%), ethanol (C_2_H_5_OH) (Merck, 96%), filter paper, and distilled water.

### 2.2. Characterization Methods

The UV–visible spectrum was recorded using a Hitachi (U–3000) spectrophotometer. The X-ray diffraction (XRD) patterns of the adsorbents were defined on a Bruker, CCD-Apex instrument by Cu-Kα radiation (λ = 1.5418 Å). Fourier–transform infrared (FT–IR) spectroscopy was performed using a Bruker spectrophotometer in the spectral range of 4000–400 cm^−1^. The morphological analysis was performed using transmission electron microscopy (TEM) (JEOL/JEM-2010). The surface and morphological characterization of the samples was performed with a scanning electron microscope (SEM; Hitachi S-4700). Their thermal stability was analyzed through thermogravimetric analysis (TGA) using a Hitachi (STA-7200) instrument. The Brunauer–Emmitt–Teller (BET) measurements were performed to determine the specific surface area and pore volume on AutoSorb (6-Quantachrome, Boynton Beach, FL, USA) [10,19]. The adsorption temperature was controlled using an Alpha Immersion thermostat (230 V, 50/60 Hz, Germany) and the initial pH was measured using a portable PHYWE pH meter (Cobra, Bremen, Germany).

### 2.3. PAni:PPy@SiO_2_ Hybrid Adsorbent Preparation

**Scheme 1:**
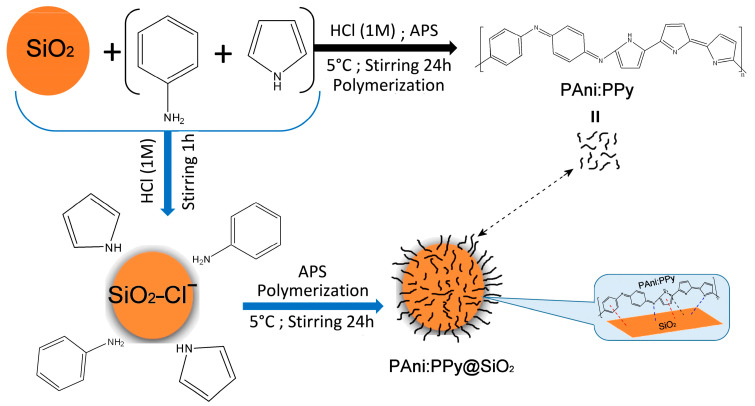
Preparation of the PAni:PPy and PAni:PPy@SiO_2_ samples.

Polyaniline:polypyrrole (PAni:PPy) was prepared by in situ chemical oxidation polymerization in 1 M HCl. The Ani and Py monomers were oxidized by APS in the presence of SiO_2_. The constant mole ratio of monomers (Ani and Py) and oxidant was n(Ani):n(Py):n(APS) = 1:1:1, the weight ratio of m(PAni:PPy):m(SiO_2_) was 90:10 for all prepared samples. The polymerization procedure was carried out in a stirred, cooled (5 °C) reactor. The APS solution was added dropwise to the monomer solution and the reaction took 24 h (Scheme 1) [10,19]. The reactor contents were then vacuum filtered and washed repeatedly with 1 M HCl, C_2_H_5_OH, and H_2_O to eliminate excess oxidant with oligomers until the filtered solution became clear. The precipitates were then dried in an oven at 60 °C for 3 h. In the same way, PAni:Ppy was prepared in the absence of SiO_2_, considering a yield of 70–73% of the adsorbents’ chemical polymerization.

### 2.4. Batch Adsorption Experiments

The adsorption experiment was carried out for the adsorption of 2,4-DCP from an aqueous solution. An amount of 25mL of 2,4-DCP was taken in 100mL flasks with a concentration of 10–200 mg·L^−1^. The adsorption test was carried out at a pH (2−12) that was adjusted by NaOH or HCl and the sample was placed in a shaker for different time periods (5–180 min) at 150 rpm in a thermal bath from 25 to 50 °C. The adsorption was determined by a UV–visible spectrophotometer at 285 nm. The adsorption efficiency of 2,4-DCP was calculated using an equation [20].

(1) $$q_{eq}=\frac{C_{0}-C_{eq}}{w}$$

where $q_{eq}$ (mg·g^−1^) denotes the adsorption capacity, *w* (g·L^−1^) is the adsorbent dose of 2,4-DCP solution, $C_{0}$ (mg·L^−1^) is the initial concentration of 2,4-DCP in contact with the adsorbent, and $C_{eq}$ is the 2,4-DCP concentration (mg·L^−1^) after the adsorption and time (*t*).

The adsorption kinetics of the 2,4-DCP were investigated at 25 °C, the adsorbent dose was introduced into 100 mL of 2,4-DCP ($C_{0}$ = 50 mg·L^−1^) for 4 h, and then the filtration and washing were performed. The experimental data were analyzed using the following models [18].

$$\text{Pseudo-first-order}\;\mathrm{formula}\;(\mathrm{PFO})\;\;\;\;{log(q}_{eq}-q_{t})={\log q}_{eq}-\frac{k_{1}}{2.303}t\;\text{Pseudo-second-order}\;\mathrm{formula}\;(\mathrm{PSO})\;\;\;\;\;\;\frac{t}{q_{t}}=\frac{1}{k_{2}q_{eq}^{2}}+\frac{1}{q_{1}}t\;\mathrm{Intraparticle}\;\mathrm{diffusion}\;(\mathrm{ID})\;\;\;\;\;q_{t}=k_{i}t^{0.5}+C$$

where, $k_{1}$ (min^−1^): PFO rate constant; $q_{t}$ (mg·g^−1^): 2,4-DCP amount adsorbed at time *t*; $q_{eq}$ (mg·g^−1^): adsorption capacity at equilibrium; $k_{2}$ (g·mg^−1^·min^−1^): PSO constant; $k_{i}$ (g·mg^−1^·min^−1^): intraparticle diffusion rate constant; and *C*: the value maximum adsorption amount.

The adsorption isotherm can be explained as the process of mass transfer between a solid and an adsorbate at a constant temperature. There are many adsorption models such as Langmuir, Freundlich, and Temkin [21]. They are expressed by:

$$\mathrm{Langmuir}\;\mathrm{equation}\;\;\;\;\;\;\frac{C_{eq}}{q_{eq}}=\frac{1}{K_{l}C_{m}}+\frac{C_{eq}}{q_{m}}$$

where $q_{eq}$ (mg·g^−1^): the amount adsorbed at equilibrium; $C_{eq}$ (mg·L^−1^): the equilibrium concentration; $K_{l}$ (L·mg^−1^): the Langmuir constant; and $q_{m}$ (mg·g^−1^): the maximum amount adsorbed.

$$\mathrm{Freundlich}\;\mathrm{equation}\;\;\;\;\;\;lnq_{eq}=lnK_{f}+\frac{1}{n}lnC_{eq}$$

where $K_{f}$ (mg^1−1/n^·g^−1^·L^1/n^): the constant of Freundlich; and *n*: the heterogeneity factor. The adsorption efficiency is connected to the $K_{f}$ constant, whereas the adsorption is related to the $\frac{1}{n}$ constant.

$$\mathrm{Temkin}\;\mathrm{equation}\;\;\;\;\;\;\;q_{eq}=Bln{(K}_{T}C_{eq})$$

where B (kJ·mol^−1^): the Temkin constant of the adsorption heat; and $K_{T}$ (L·mg^−1^): the constant of the Temkin isotherm.

$$\mathrm{Sips}\;\mathrm{equation}\;\;\;\;q_{eq}=q_{m}\frac{K_{s}C_{eq}n_{s}}{1+K_{s}n_{s}}$$

where $K_{s}$ (L·g^−1^): the Sips constant; and $n_{s}$ (L·g^−1^): the Sips isotherm exponent.

$$\mathrm{Redlich}–\mathrm{Peterson}\;\;\;\;q_{eq}=\frac{K_{RP}C_{eq}}{1+a_{RP}C_{eq}\beta }$$

where $K_{RP}$ (L·g^−1^): the Redlich–Peterson constant; and β: the Redlich–Peterson isotherm exponent developed to overcome the disadvantage of the Freundlich model. At high and low adsorbate concentrations, the Sips isotherm approaches the Langmuir and Freundlich isotherms [22], respectively. The equation of the Sips isotherm is given below.

The root mean square error (RMSE) functions were adopted to optimize the model parameters, which can be expressed as:

$$RMSE=\sqrt{\frac{1}{m-p}}\sum_{i=1}^{m}{\left(q-q_{eq}\right)}^{2}$$

where m: the number of data points evaluated; and p: the number of parameters in the regression model.

Thermodynamic equations were used to determine the change in enthalpy (ΔH), the change in free energy (${\Delta G}^{0}$), and the change in entropy (${\Delta S}^{0}$) for 2,4-DCP adsorption on the adsorbents. These variables are used to determine the sorption type [23]. $K_{D}$ defines the equilibrium constant, which can be measured by the following equations: $K_{D}=\frac{q_{eq}}{C_{eq}}$

$$\mathrm{Free}\;\mathrm{enthalpy}\;\;\;\;\;\;\;{\Delta G}^{0}=-RTln\left(K_{D}\right)$$

where *T* (K): absolute temperature; *R* (8.314 J·mol^−1^·K^−1^): ideal gas constant; and $K_{D}$: the distribution coefficient.

The ${\Delta H}^{0}$ and ${\Delta S}^{0}$ values can be measured from the Van’t Hoff formula:

$$\;\;\;\;\;\;\;\;\;{lnK}_{D}=\frac{{\Delta G}^{0}}{RT}=-\frac{{\Delta H}^{0}}{RT}+\frac{{\Delta S}^{0}}{R}\;K_{D}=\frac{q_{eq}}{C_{eq}}$$

## 3. Results and Discussion

### 3.1. Characterization Analysis

**Figure 1:**
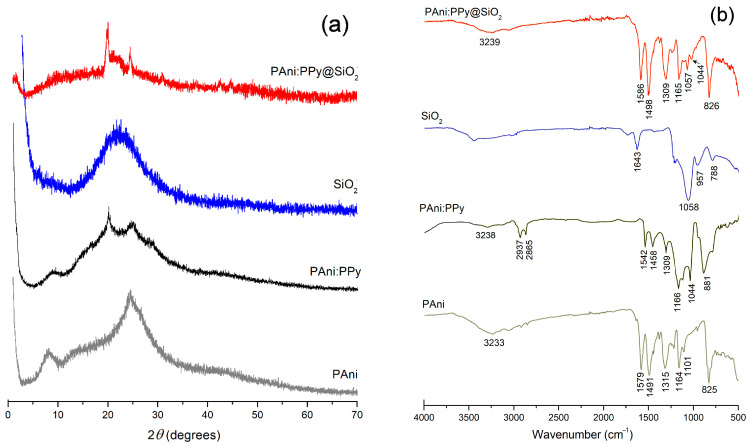
(**a**) XRD pattern and (**b**) FTIR spectrum of adsorbent samples.

The XRD patterns of the as-synthesized PAni, PAni:PPy, SiO_2_, and PAni:PPy@SiO_2_ are shown in Figure 1a. PAni has a semi-crystalline structure, as the patterns show two peaks at 2*θ* = 8.21° and 24.86°, which are attributed to the presence of quinonoid with benzenoid functions in the polymer backbone [19]. Moreover, the XRD patterns of PAni:PPy show a relatively intense band at 2*θ* = 19.95° with two distinct shoulders at 8.34° and 25.05°. The observed peak at 2*θ* = 25.05° might be due to the overlapping between the prevailing (110) reflection of PPy and the peak associated with the PAni. For the SiO_2_ sample, a peak at 2*θ* = 22.41° is observed, which can be attributed to the amorphous silica (SiO_2_, JCPDS No. 29-0085) [24]. No other peaks can be found. In the case of the PAni:PPy@SiO_2_ adsorbent, aside from the characteristic peak of SiO_2_, all other XRD peaks can be indexed to the PAni:PPy sample, indicating that the PAni:PPy matrix was formed on the SiO_2_ surface. This indicates that reactions occurring between the SiO_2_ and the polymer chains occur during its preparation.

Figure 1b represents the FTIR spectra of the samples. The spectra for PAni show the characteristic band of nitrogen quinine at 1579 cm^−1^. Bands at 1491 cm^−1^, 1315 cm^−1^, 1164 cm^−1^, and 1101 cm^−1^ appear, which are associated with a benzene ring and C−N and C=N stretching vibrations. In addition, the band at 3233 cm^−1^ is associated with a C–H stretching vibration. The peak at 825 cm^−1^ belongs to the C−H of the PAni ring. Compared to PAni, the bands of PAni:PPy are redshifted. This may be attributed to the strong interactions between the copolymer backbone during the synthesis process [24,25,26], such as π-π stacking or H bonding between nitrogen and hydrogen. The typical spectral bands of PAni and PPy can be observed in the copolymer spectra, indicating that the pyrrole and aniline monomers are present in the copolymer structure. These indicate that the core-shell structure copolymer was successfully prepared and it presents the conductive state, which facilitates the transfer of electrons collected between them. The FTIR spectrum of SiO_2_ displayed the presence of symmetric and asymmetric stretching modes for Si−O, Si−OH, and Si−O−Si groups at around 1058 cm^−1^, 957 cm^−1^, and 781 cm^−1^, respectively. In addition, there is a broad band located around 3400 cm^−1^ and another located at 1634 cm^−1^, both attributed to the O–H stretching of H_2_O and the formed silanol groups (Si–OH), respectively [27]. The interactions between the PAni:PPy structure and SiO_2_ resulted in a slight redshift of the C=C and C=N bonds from 1542 cm^−1^ and 1458 cm^−1^ for copolymer alone to 1586 cm^−1^ and 1498 cm^−1^ for PAni:PPy@SiO_2_ in the hybrid adsorbent. This confirmed the modification of the PAni:PPy matrix by SiO_2_.

**Figure 2:**
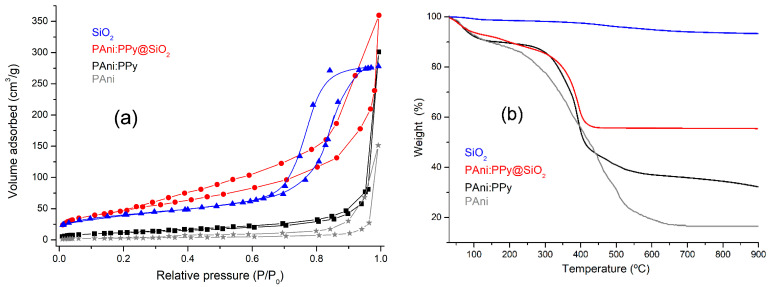
(**a**) BET surface area and (**b**) TGA curves of material samples.

The Brunauer–Emmitt–Teller (BET) method was used to investigate the BET surface areas, pore volumes, and pore size distributions of the samples (Figure 2a), and the results are summarized in Table 1. Clearly, the N_2_ adsorption–desorption isotherms of all the materials belonged to type III with H_3_ hysteresis loops, indicating the mesoporous structures in these samples [27]. The typical BET-specific surface area of SiO_2_ is rather higher (182.02 m^2^·g^−1^) compared to that of the PAni:PPy@SiO_2_ material (113.84 m^2^·g^−1^). The reduction in the surface area is attributed to the PAni:PPy matrix blocking the pores of the SiO_2_, thus reducing the surface area [27].

TGA is widely used to study the thermal degradation of materials [9]. The technique analyzes the weight loss associated with a continuous increase in temperature. The TGA lines represent the thermal degradation behavior of different synthesized materials in Figure 2b. The thermal treatment of the PAni:PPy@SiO_2_ composite consists of three levels of weight loss (wt). The first (wt) level up to 110 °C is attributed to the volatilization and decomposition of small organic compounds of 5.27%. The second (wt) level is up to 9.95% at 300 °C due to the evaporation of absorbed moisture and residual monomer or oligomer loss and the following third (wt) level reached up to 420 °C due to the chain degradation and decomposition of the material being 29.03%. In addition, a steady state decrease in weight of 11.91% of the PAni:PPy was observed up to 300 °C. Then, a steep decreasing trend in weight was observed up to 37.88% at 410 °C. After this, a steady state was again observed starting from 430 °C up to 900 °C. Moreover, the 13.46% weight of PAni was exhausted at 220 °C, a lower temperature than the PAni:PPy copolymer. Following a similar trend, the 68.89% weight was exhausted at about 630 °C. Finally, the SiO_2_ shows initial (wt) corresponding to the evaporation of water followed by steady (wt) up to 900 °C due to the final loss caused by the decomposition of the component with residual mass retained being 6.61%.

**Figure 3:**
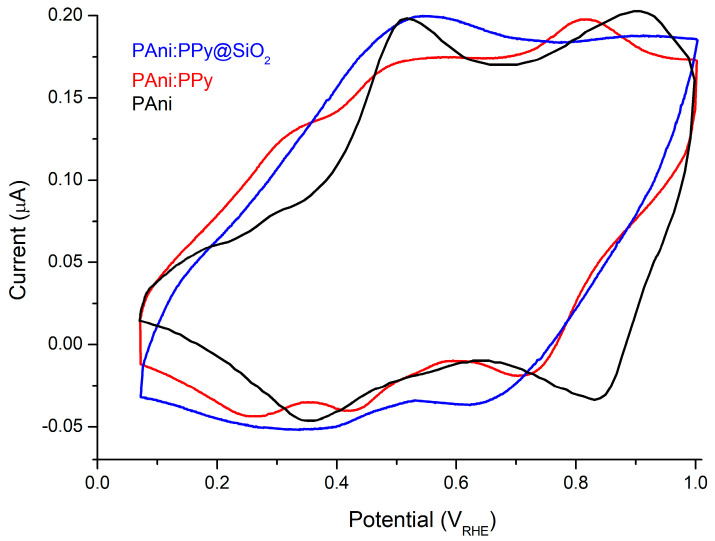
Cyclic voltammograms recorded for a carbon electrode covered with adsorbent samples in HCl solution (1 M) at a scan rate of 50 mV·s^−1^.

The CV curves of PAni, PAni:PPy, and PAni:PPy@SiO_2_ are shown in Figure 3. For the pristine PAni, two oxidation–reduction peaks were observed and these peaks are due to redox reactions with H^+^ ions in the electrolyte. The oxidation peak at 0.46 V and the corresponding reduction peak at 0.29 V are attributed to the redox reaction from the leucoemeraldine form to the protonated emeraldine form. In addition, the redox peaks with higher oxidation states (0.89/0.81 V) can be associated with the transition of emeraldine to the totally oxidized pernigraniline [10]. It is noted that the PAni:PPy sample can show good electrochemical performance. Moreover, its CV curve has three pairs of redox peaks at (0.33/0.26 V), (0.50/0.42 V), and (0.81/0.71 V), indicating the copolymer formation. For the CV curve of the PAni:PPy@SiO_2_ sample, the oxidation sweep peaks were at 0.50 V and the reduction peaks appeared at almost 0.67 V and 0.38 V. An apparent shift of the peaks was found between PAni and copolymer, which may be due to the formation of SiO_2_ on the matrix polymer.

**Figure 4:**
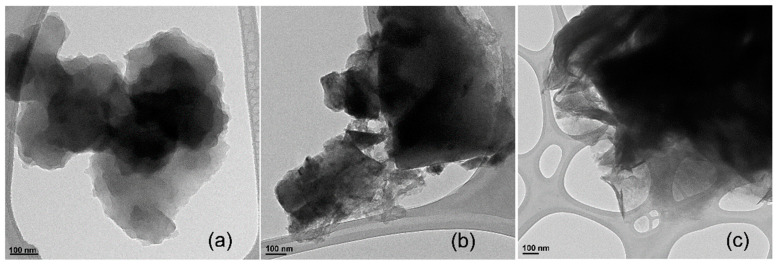
TEM images of (**a**) PAni, (**b**) PAni:PPy, and (**c**) PAni:PPy@SiO_2_.

The morphological characteristics of the materials synthesized were studied by TEM. Figure 4 exhibits the TEM images for PAni, PAni:PPy, and PAni:PPy@SiO_2_. The first conspicuous difference between the PAni and the copolymer that can be observed in the images is that the degree of aggregation is higher in the copolymer compared to PAni. In addition, the TEM image of the PAni:PPy@SiO_2_ shows that the material has a very dense and packed structure. Due to their high surface area and energy, the SiO_2_ nanoparticles may have aggregated on the PAni:PPy matrix. An outer shell corresponding to the copolymer formed can be seen, explaining the observation of a darker surface area.

**Figure 5:**
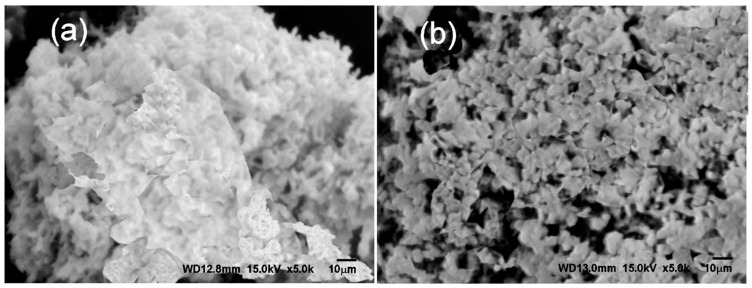
SEM images of (**a**) PAni:PPy and (**b**) PAni:PPy@SiO_2_.

SEM was used to investigate the surface morphology of PAni:PPy and PAni:PPy@SiO_2_. Figure 5a shows that the copolymer has a relatively amorphous nature and the chain structures are well interconnected. Likewise, Figure 5b is the SEM image of PAni:PPy@SiO_2_ which shows that SiO_2_ was dispersed homogeneously and buried inside the PAni matrix with a random stacking morphology.

**Figure 6:**
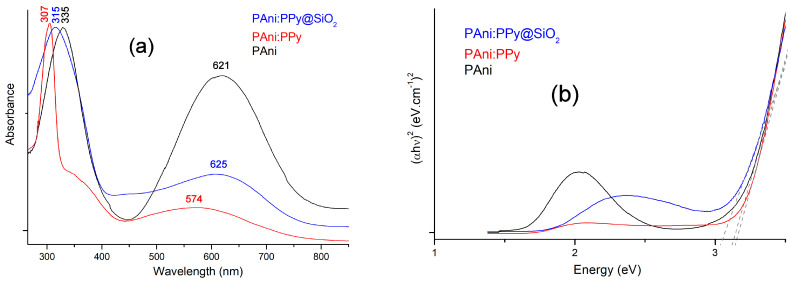
(**a**): UV–vis absorption spectra and (**b**) Tauc plots of adsorbent samples.

Figure 6a shows the UV–vis spectra of pure PAni, PAni:PPy, and PAni:PPy@SiO_2_. Two absorption bands were observed near 307–335 nm and 574–625 nm. The first discovered band is assigned to the π–π* transition for the pyrrole and/or benzenoid ring [28]. The second band is assigned to the pyrrole and/or quinoid ring transition [28]. These absorption bands provide general information about the oxidation/reduction state of the polymer backbone.

The effect of the presence of SiO_2_ on the optical properties and hence the optical energy band gap ($E_{g}$) of the polymer matrix was determined. Tauc’s relation was used to determine the $E_{g}$ values of the samples (Figure 6b). In addition, the $E_{g}$ of the samples is derived by the extrapolation of the linear part of the (*ahν*) curves to fit the (hν) axis.

$$\left(ah\nu {)}^{n}=B(h\nu -E_{g}\right)$$

where hν: the energy of the incident photon; a: the absorption coefficient; B: constant; $E_{g}$: the optical band gap (eV); and *n* is a variable exponent (n=1/2 when the transition is indirect and n=2 when the transition is direct) [29].

It was found that the band gap energy for PAni:PPy@SiO_2_ is 3.15 eV; it then decreases to 3.13 eV (for pure PAni) and 3.05 eV (for copolymer PAni:PPy). The shrinkage of the optical band gap is mainly attributed to localized states created in the energy band gap of the SiO_2_ material. Tailoring the optical properties and hence the optical band gap of PAni:PPy@SiO_2_ makes it eligible for new applications.

### 3.2. Adsorption Experiences

#### 3.2.1. Effect of Key Factors

The point of zero charges (pH_PZC_) can be formed when the pH surface charge tends to zero, and such pH_PZC_ is employed to define the electrostatic interaction between the adsorbent surface and adsorbate. For the determination of the pH_PZC_ value, 0.05 g of each adsorbent was mixed for 24 h at room temperature with 50 mL of 0.1 M NaCl solution. A NaOH and HCl solution (0.01 M) was used to adjust the pH between 2 to 12. When the pH is less than pH_PZC_, the surface of the adsorbent is expected to be negatively charged and it allows the adsorption of positive ions. At a pH greater than pH_PZC_, the adsorption of anions is more favorable due to increased positive ions at the adsorbent surface [30].

**Figure 7:**
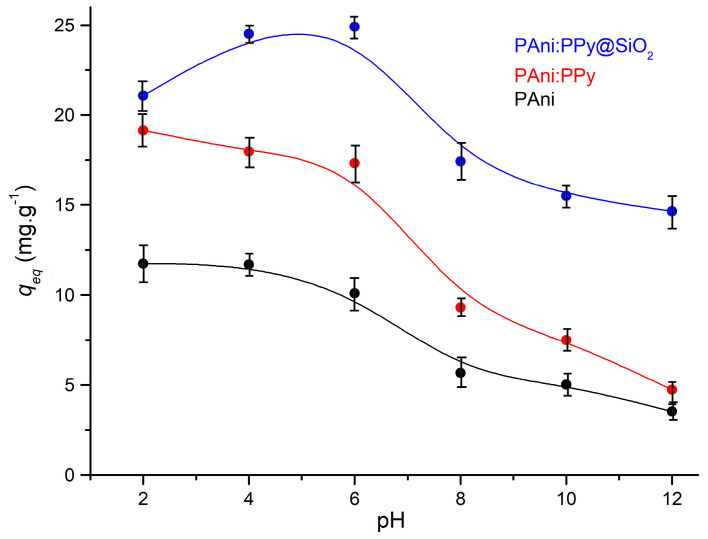
Effect of pH on the adsorption capacity (*C*_0_: 50 mg·L^−1^; T: 298 K; adsorbent dose: 0.05 g).

The pH not only affects the specific adsorption and the charge on the adsorbent surface but also influences the solubility of the phenolic compounds [3,31]. Therefore, the adsorption percentage changes when the pH of a solution is varied. The effect of the initial pH value was investigated by varying the pH from 2 to 12 on the adsorption of 2,4-DCP by the three adsorbents, and the results are shown in Figure 7. It is clear that the adsorption capacity of PAni:PPy@SiO_2_ increased gradually with increasing pH from 2.0 to 6.0 and then greatly decreased at pH 12.0. Meanwhile, pH values can significantly affect the surface charges of the adsorbate and thus the protonation level of functional groups on its surface [32]. The surface charge (pH_zpc_) of PAni:PPy@SiO_2_ was measured to be 8.5. This signifies that at pH > 8.5, the charge on the PAni:PPy@SiO_2_ surface is negative, whereas, at pH < 8.5, this charge is positive. Furthermore, the maximum *q_eq_* of the PAni:PPy@SiO_2_ was 24.9 mg·g^−1^ reached at pH 6.0, which is comparable to the removal of 2,4-DCP adsorbate on silicate adsorbent, where the am value was 2.16 mg·g^−1^ [30]. This was 7.43 mg·g^−1^ for PAni/barley husk (PAni/BH) and 9.73 mg·g^−1^ for PPy/barley husk (PPY/BH) adsorbent [3]. As the pH increased to 12.0, the $q_{eq}$ of the adsorbent decreased sharply to 14.0 mg·g^−1^. Moreover, it is well known that the pK_a_ value of 2,4-DCP is 7.90 [33], and its molecules favor a hydrophilic form when pH > pK_a_. More specifically, in the medium with a higher pH value (from 6.5 to 8.5), the removal rate rapidly decreased due to the ionization and the hydrophilic behavior of the chlorophenols. Although there is a π−π interaction and electrostatic attraction between the positive surface of the adsorbent sites and the anionic 2,4-DCP species, it plays only a secondary role in the removal process because the anionic 2,4-DCP molecules could not form H bonds and inclusion complexes with PAni:PPy@SiO_2_ in a higher pH solution. On the other hand, at pH > 8.5, the adsorption capacity is significantly decreased due to the repulsion between the adsorbent surface and the negative charge of the 2,4-DCP molecules. As it is known, the adsorbate is mainly in a protonated form at pH < pK_a_ (7.90) and in a deprotonated form at pH > pK_a_. Therefore, a pH of 6.0 was chosen for the present study. Furthermore, the pH_pzc_ was found to be 4.0 in the case of PAni and PAni:PPy [2]. The removal capacity (*q_eq_*) of 2,4-DCP by these adsorbents decreased with the increase in pH. As can be seen, the trend of $q_{eq}$ over pH in the range of 2–6 was typically the same as the trend of 2,4-DCP adsorption using treated PAni and PAni:PPy, which is due to the excess of H^+^ ions competing with the adsorbate for active surface sites. However, when the pH was increased to 12, the adsorption capacity decreased to 4.73 mg·g^−1^ (PAni:PPy) and 3.53 mg·g^−1^ (PAni), respectively.

**Figure 8:**
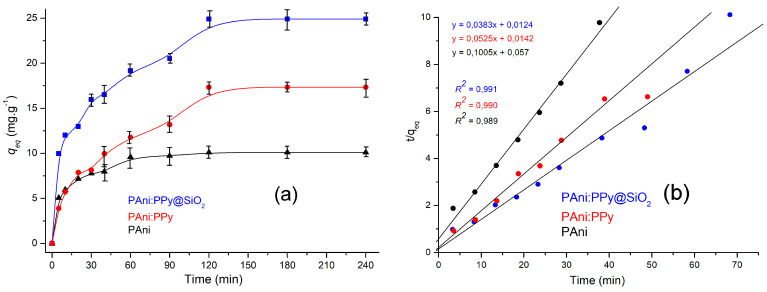
(**a**) Contact time (*C*_0_: 50 mg·L^−1^; *pH*: 6.0; T: 298K; adsorbent dose: 0.05 g). (**b**) The experimental kinetic data were fitted by the PSO kinetic model.

The contact time greatly affects the adsorption capacity of the adsorbent, and the response is depicted in Figure 8a. Obviously, the $q_{eq}$ value increased sharply within 5 min due to the sufficient mass transfer stimulus and became slower with time, and then the adsorption reached equilibrium at 120 min. Thereafter, there was no significant change in the adsorption rate. Therefore, the optimum contact time to obtain the $q_{eq}$ was observed to be 120 min for the three adsorbent materials. The cause of such adsorption behavior of 2,4-DCP may be due to the accessibility of active sites on the adsorbent; mainly, the active sites were readily available for adsorbent binding, which saturated with time and then the adsorbate progressively started to move inwards, leading to the slow adsorption capacities [32]. The slow adsorption rate in the subsequent phases might be due to the intramolecular diffusion mechanism [34]. Similar behavior has been studied previously: as the contact time progresses, adsorption may decrease due to the saturation of binding sites [3,34].

To understand the adsorption-rate-determining step, the PFO, PSO, and intraparticle diffusion (ID) models were used to describe the 2,4-DCP adsorption process by three ad-sorbent samples [35]. Furthermore, adsorption kinetics data were fitted by the PSO model (Figure 8b). Interestingly, Table 2 lists the measured values for different adsorption kinetic models. The coefficient of determination (*R*^2^) and the root mean square error (RMSE) were used as error parameters for each model. The results indicate that the PSO model had the lowest values for the error parameters RMSE and the highest *R*^2^ values. In addition, the $q_{eq}$ values calculated for the PSO are in good agreement with the experimental findings. Thus, it can be said that the PSO model gave the best approximation of the kinetics of 2,4-DCP adsorption by the adsorbents than the PFO and ID models.

#### 3.2.2. Adsorption Isotherms

**Figure 9:**
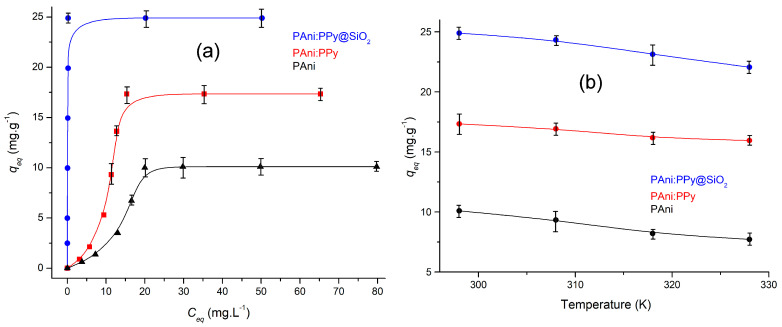
(**a**) Adsorption isotherms (adsorbent dose: 0.05 g; 2,4-DCP: 50 mL; T: 298 K; *pH*: 6.0). (**b**) Influence of temperature on elimination percentage (adsorbent dose: 0.05 g; 2,4-DCP: 50 mL; *pH* 6.0).

Adsorption isotherms provide an understanding of the elimination process. The adsorption mechanism of the adsorbent consists of three steps. First, the 2,4-DCP molecules are transferred from the aqueous solution to the adsorbent surfaces via a liquid boundary film. Next, these adsorbates are transferred from the adsorbent surface to the intramolecular binding site, and finally, there is a strong attraction of the 2,4-DCP with the disposable sites both on the external surface and internal surface of the adsorbents [10]. Figure 9a shows the development of the adsorbed quantity as a function of 2,4-DCP concentration at equilibrium. The figure depicts that the adsorbed amount of 2,4-DCP increases with the concentrations. This is defined by the ease of adsorbent–adsorbate mass transfer [36]. Furthermore, the maximum adsorption efficiency value of PAni:PPy@SiO_2_ was 24.9 mg·g^−1^, which is approximately 1.5 times higher than that of PAni:PPy (17.34 mg·g^−1^) and 2.5 times higher than that of PAni. The difference in the adsorption capacity indicates that the formation of SiO_2_ on the copolymer matrix drastically enhanced the adsorption process. The adsorption capacity of PAni:PPy@SiO_2_ was almost the highest value among the adsorbents reported in previous studies for 2,4-DCP (Table 3). Therefore, PAni:PPy@SiO_2_ can be an efficient and promising adsorbent material for the removal of organic pollutants.

The results of the adsorption isotherm results are summarized in Table 4. As can be seen from (*R*^2^), the values determined for the Freundlich and the Temkin models are less favorable, suggesting that these isotherm models cannot adequately describe this adsorption process. Otherwise, the adsorption isotherm can be better described by the Langmuir and combined models, and the measured adsorption capacity is comparable to the empirical one, suggesting that 2,4-DCP removal on these adsorbents is a monolayer homogeneous adsorption. Furthermore, the PAni:PPy@SiO_2_ results offer a better fit by the Temkin model with a value of *R*^2^ = 0.984 compared to the Freundlich isotherm (*R*^2^ = 0.881), suggesting that the adsorption energy decreases linearly as the binding sites are saturated by the 2,4-DCP. Furthermore, the Langmuir constant ($K_{L}$) represents the intensity of the adsorbent/adsorbate interaction [41]. Here, it was noted that its value is 0.843 L·mg^−1^. The latter is relatively higher compared to the PAni and PANI:PPy adsorbents, indicating a strong interaction between 2,4-DCP and the PAni:PPy@SiO_2_ surface. Furthermore, the n value related to the Freundlich isotherm (*n* > 1) confirmed that the PAni:PPy@SiO_2_ material was suitable for 2,4-DCP absorption. On the other hand, the *R*^2^ values were weaker than those measured from other isothermal kinetics. Therefore, Langmuir’s isotherm is in complete agreement with the empirical results for this adsorbate on the PAni:PPy@SiO_2_ surface. Likewise, the measured results suggested that the as-prepared PAni:PPy and PAni exhibited different adsorption behavior on 2,4-DCP. The data of these adsorbents were well approximated by the Langmuir and Freundlich models with the highest regression coefficients *R^2^*, respectively. The Freundlich isotherm is better at describing the 2,4-DCP elimination by polymer and copolymer compared to PAni:PPy@SiO_2_, which may be due to the specific surface areas, different morphology, composition, and structural arrangements of these adsorbents. On the other hand, the fitted parameter β in the Redlich–Peterson (R-P) model was between 0.90 and 0.95, indicating that the R-P model could be converted into the Langmuir model in these cases and that the adsorption sites on these adsorbents were on a single layer, and the adsorbed 2,4-DCP had no internal interaction [21]. In addition, the Sips model was well-fitted to 2,4-DCP adsorption data with *R*^2^ values greater than 0.996, suggesting the heterogeneous surface adsorption of 2,4-DCP on adsorbents [10].

### 3.3. Adsorption Thermodynamics

The effect of temperature on the 2,4-DCP adsorption was studied by changing the temperature of the adsorbent from 298 K to 328 K, as shown in Figure 9b. A decrease in removal productivity was observed with the increase in temperature, which could indicate that an increase in temperature participates in a decrease in adsorption capacity on the three adsorbent samples. These results showed the exothermic character of 2,4-DCP elimination by adsorbents. This adsorption behavior is attributed to the inactivity of active sites at high temperatures [3]. The measured thermodynamic data are summarized in Table 5. The ${\Delta H}^{0}$ negative value suggests that the process of adsorption at all investigated temperatures is exothermic in nature. Negative values of Δ*S* were obtained, showing that there is a decrease in the disorderliness and haphazardness of the adsorbed molecules’ movement at elevated temperatures. Moreover, there were decreasing negative values of ${\Delta G}^{0}$ in with increasing temperature, signifying that the process and capacity for the adsorption reduce with the increasing temperature; this process is spontaneous, thermodynamically feasible, and chemically controlled [31]. When the values of thermodynamic parameters for the adsorption of the 2,4-DCP solution onto the PAni:PPy@SiO_2_ were compared with the polymer adsorbents, higher absolute values of change in the ${\Delta G}^{0}$ as well as the ${\Delta H}^{0}$ was observed. Precisely, the change in ${\Delta H}^{0}$ for the adsorption by PAni:PPy@SiO_2_ was obtained as −94.76 kJ·mol^−1^ compared to −12.08 kJ·mol^−1^ and −7.15 kJ·mol^−1^ for PAni:PPy and PAni, respectively, indicating the tendency for higher adsorption capacity in presence of SiO_2_. Importantly, the results also indicate that ${\Delta H}^{0}$ lower than 20 kJ·mol^−1^ denotes that the adsorption mechanism is physisorption (the bond between adsorbent and adsorbate are hydrogen bonds or van der Waals interactions); in addition, physisorption is involved in the process when ${\Delta G}^{0}$ ranges from −20 < ${\Delta G}^{0}$ < 0 kJ·mol^−1^ [21,25].

### 3.4. Adsorption Mechanism

Based on the above experimental results and discussions, the proposed adsorption mechanism of 2,4-DCP adsorption on the three adsorbents from aqueous solution is presented as follows.

**Scheme 2:**
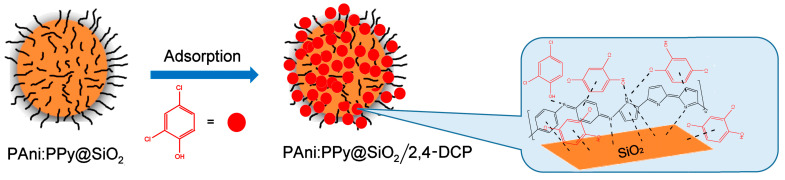
Proposed adsorption mechanism of 2.4-DCP by PAni:PPy@SiO_2_.

The adsorption mechanism of 2,4-DCP on PAni and PAni:PPy chain can be attributed to several forms of interactions [3]. The mechanism involves the electrostatic interaction between negatively charged groups available on the adsorbent surface with positively charged groups of the 2,4-DCP. The adsorption mechanism also includes hydrogen bonding interactions between nitrogen atoms in the polymer backbone and hydrogen atoms in the 2,4-DCP molecule. Finally, π–π interactions can occur between the aromatic rings of the polymer matrix and the aromatic ring of 2,4-DCP. According to the possibilities mentioned above, these interactions were responsible and essential for the enhancement of the 2,4-DCP adsorption on the PAni and PAni:PPy surfaces. Similar results have been reported by other studies for the adsorption of 2,4-DCP by biocomposites [3] and herbicide by polyaniline/polypyrrole composites with cellulosic biomass [2]. In the case of the PAni:PPy@SiO_2_ adsorbent, we know that the 2,4-DCP is a positively charged molecule that can interact electrostatically with negatively charged molecules for SiO_2_ moieties and also with electron-rich moieties in the polymer backbone. In addition, the formation of hydrogen bonds between nitrogen atoms for copolymer (PAni:PPy) and hydrogen-containing groups of 2,4-DCP can also occur. The π–π interactions between the aromatic rings of the adsorbate molecules and the copolymer chain structures of the adsorbent can be expected. Scheme 2 shows the proposed adsorption mechanism of adsorbents of 2.4-DCP by PAni:PPy@SiO_2_.

### 3.5. Reusability of Adsorbents

**Figure 10:**
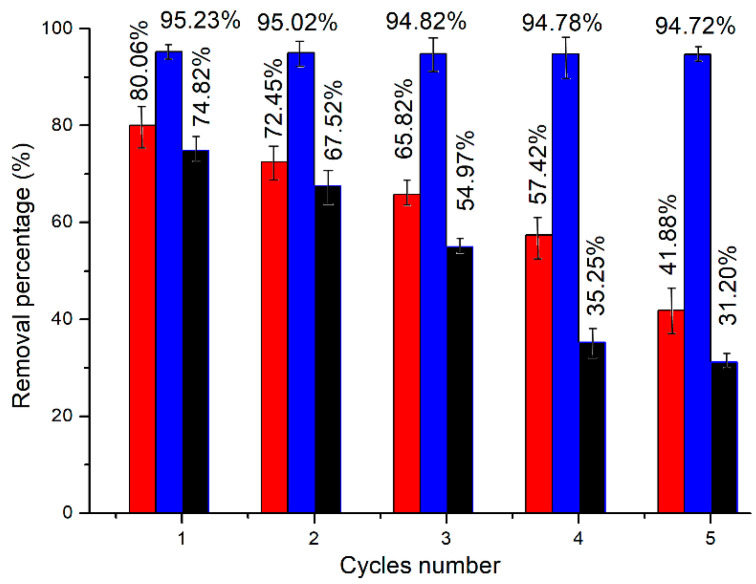
Cyclic adsorption–desorption performance of adsorbents (Black: PAni; Red: PAni:PPy and Blue: PAni:PPy@SiO_2_) on 2,4-DCP (adsorbents dose: 0.05 g; 2,4-DCP: 50 mL; T: 298 K; *pH* 6.0).

Adsorbent regeneration was verified, defined as the ability to recover the 2,4-DCP molecules accumulated on the surface of the adsorbent and make it feasible to reuse in further adsorption/desorption cycles. The adsorbent/adsorbate interaction force and the amount of adsorbate loaded on the adsorbent play an important role in the regeneration test. Therefore, in the above context, the response surface optimized conditions were pH 6.0, 0.1 g/100 mL of adsorbent dosage, and an initial 2,4-DCP concentration of 50 mg·L^−1^, followed by elution with 0.1 M HNO_3_. Five successive adsorption–desorption experiments were performed to measure the reusability of the adsorbents. After each experiment, the adsorbents were washed with distilled water, then dried at 60 °C, and reused for the next run. Figure 10 exhibits the materials’ reusability for 2,4-DCP removal. It can be observed that PAni:PPy@SiO_2_ maintains a very significant level of 2,4-DCP adsorption during five cycles, thus emerging as a typical alternative to traditional adsorbents for the elimination of this class of pollutants. Conversely, it should be noted that polymer and copolymer showed a decrease in productivity after each cycle. The percentages of adsorption of 2,4-DCP by PAni:PPy for the second, third, fourth, and fifth cycles were 72.45%, 65.82%, 57.42%, and 41.88%, respectively Such a remarkable decrease is due to the fundamental weight loss that took place at the time of regeneration and during the washing of the adsorbent. This also leads to a decrease in adsorption capacity in every regenerated cycle because of the reduction in binding sites present during the process owing to the 2,4-DCP molecules that were adsorbed. The results showed that about 41% of 2,4-DCP was eliminated in the fifth cycle, which is a significant rate, indicating the higher adsorption capacity of PAni:PPy adsorbent. In addition, after the first two runs, PAni progressively reduced its ability to adsorb 2,4-DCP, which is related to a gradual saturation of the active sites, even though its adsorption capacity remained greater than 31% after five cycles.

## 4. Conclusions

In this work, a simple method was used to prepare a novel adsorbent of PAni:PPy@SiO_2_, and its effects on the elimination of 2,4-DCP were studied. This adsorbent was formed by the in situ polymerization of pyrrole and aniline in the presence of SiO_2_ by APS. In order to determine the composition and electrochemical behavior of the samples, their optical and chemical compositions and morphological characteristics were determined using ultraviolet–visible spectrophotometry, X-ray diffraction, infrared spectroscopy, thermogravimetric analysis, and transmission electron microscopy. Moreover, the PAni:PPy@SiO_2_ obtained a significantly high removal capacity of 24.90 mg·g^−1^ at pH 6.0, T = 25 °C, [2,4-DCP] = 50 mg·L^−1^, and a homogeneous chemical monolayer adsorption process was described according to the PSO and Langmuir isotherm models. The enthalpy change (ΔH), free energy change (ΔG), and entropy change (ΔS) data were also determined, which showed the favorable exothermic adsorption process of 2,4-DCP. Furthermore, the PAni:PPy@SiO_2_ showed perfect recyclability, which is an important advantage in the adsorption process.

## Figures and Tables

**Table 1:** Textural properties of adsorbents.

Material	S_BET_/m^2^·g^−1^	Pore Volume/cm^3^·g^−1^	Average Pore Size nm
PAni	43.18 ± 0.01	1.79 ± 0.01	28.64 ± 0.01
PAni:PPy	38.41 ± 0.01	0.98 ± 0.01	22.82 ± 0.01
SiO_2_	182.02 ± 0.01	2.21 ± 0.01	37.55 ± 0.01
PAni:PPy@SiO_2_	113.84 ± 0.01	2.05 ± 0.01	30.14 ± 0.01

**Table 2:** PFO, PSO, and ID adsorption rate constants for the elimination of 2,4-DCP by different adsorbents at 298 K, pH: 6.0, and Co:50 mg·L^−1^.

Models	Constants	PAni	PAni:PPy	PAni:PPy@SiO_2_
PFO	$k_{1}$ (min^−1^)	0.017 ± 0.02	0.020 ± 0.02	0.045 ± 0.02
	$q_{eq.Cal}$ (mg·g^−1^)	5.59 ± 0.1	14.94 ± 0.1	19.91 ± 0.1
	*RMSE*	1.82 ± 0.1	0.97 ± 0.1	1.31 ± 0.1
	*R^2^*	0.853 ± 0.02	0.939 ± 0.02	0.908 ± 0.02
PSO	$q_{eq.Exp}$ (mg·g^−1^)	10.10 ± 0.1	17.34 ± 0.1	24.90 ± 0.1
	$k_{2ads}$ (g·mg^−1^·min^−1^)	0.177 ± 0.02	0.194 ± 0.02	0.118 ± 0.02
	$q_{eq.Cal}$ (mg·g^−1^)	9.95 ± 0.1	19.04 ± 0.1	26.08 ± 0.1
	*RMSE*	0.28 ± 0.1	0.14 ± 0.1	0.11 ± 0.1
	*R* ^2^	0.989 ± 0.02	0.990 ± 0.02	0.991 ± 0.02
Intraparticle diffusion	$k_{i}$ (g·mg^−1^·min^−1^)	2.734 ± 0.02	2.186 ± 0.02	1.642 ± 0.02
	*C* (mg·g^−1^)	1.839 ± 0.02	0.920 ± 0.02	2.933 ± 0.02
	*RMSE*	2.55 ± 0.1	1.05 ± 0.1	1.93 ± 0.1
	*R* ^2^	0.729 ± 0.02	0.941 ± 0.02	0.879 ± 0.02

**Table 3:** Adsorption efficiencies of 2,4-DCP on several adsorbents.

Adsorbents	$q_{eq}$ (mg·g^−1^)	pH	$C_{0}$ (mg·g^−1^)	Ref.
Native rice husk (BH)	10.86	6.0	25	[3]
PAni/BH	24.57	6.0	25	[3]
PPy/BH	19.05	6.0	25	[3]
NaAlg/BH	7.55	6.0	25	[3]
βCD-BIMOTs-TDI	29.58	6.0	100	[31]
Activated carbon fiber	2.25	6.0	0.8	[33]
β-Cyclodextrin/attapulgite composites	19.04	8.0	100	[37]
Aged-refuse	1.53	6.0	100	[38]
β-Cyclodextrin epichlorohydrin polymer	15.7	7.0	98	[39]
γ-Cyclodextrin epichlorohydrin polymer	9.8	7.0	98	[39]
Palm pith carbon	19.16	2.0	20	[40]
PAni	10.10	6.0	50	This work
PAni:PPy	17.34	6.0	50	This work
PAni:PPy@SiO_2_	24.90	6.0	50	This work

**Table 4:** Langmuir, Freundlich, Temkin, Sips, and Redlich–Peterson isotherm constants and their correlation coefficients for the elimination of 2,4-DCP on adsorbents at pH:6.0 and 298K.

Models	Constants	PAni	PAni:PPy	PAni:PPy@SiO_2_
Langmuir	$q_{eq.Exp}$ (mg·g^−1^)	10.10 ± 0.1	17.34 ± 0.1	24.90± 0.1
	$q_{eq.Cal}$ (mg·g^−1^)	8.85 ± 0.1	18.91 ± 0.1	26.08 ± 0.1
	$K_{L}$ (L·mg^−1^)	0.237 ± 0.02	0.134 ± 0.02	0.843 ± 0.02
	$R_{L}$	0.448 ± 0.02	0.464 ± 0.02	0.083 ± 0.02
	*R* ^2^	0.993 ± 0.02	0.997 ± 0.02	0.994 ± 0.02
Freundlich	$K_{F}$ (mg^1−1/n^L^1/n^g^−1^)	32.53 ± 0.1	22.75 ± 0.1	36.29 ± 0.1
	n	0.606 ± 0.02	0.524 ± 0.02	1.405 ± 0.02
	*R^2^*	0.972 ± 0.02	0.975 ± 0.02	0.881 ± 0.02
Temkin	B (T·mol^−1^)	2.544 ± 0.02	4.065 ± 0.02	29.86 ± 0.1
	$K_{T}$ (L·g^−1^)	40.64 ± 0.1	35.07 ± 0.1	73.86 ± 0.1
	*R^2^*	0.792 ± 0.02	0.784 ± 0.02	0.984 ± 0.02
	$q_{s}$ (mg·g^−1^)	9.62 ± 0.1	18.04 ± 0.1	24.11 ± 0.1
Sips	$K_{s}$ (L·g^−1^)	0.009 ± 0.01	0.006± 0.01	0.004 ± 0.01
	$n_{s}$	2.53 ± 0.1	3.56 ± 0.	2.34 ± 0.1
	*R^2^*	0.996 ± 0.01	0.998 ± 0.01	0.997 ± 0.01
	$K_{RP}$ (L·g^−1^)	162 ± 0.9	343 ± 0.9	203 ± 0.9
Redlich–Peterson	$a_{R}$ (L·mg^−1^)	177 ± 0.9	40.8 ± 0.5	20.5 ± 0.5
	*β*	0.95 ± 0.5	0.90 ± 0.5	0.94 ± 0.5
	*R* ^2^	0.995 ± 0.02	0.997 ± 0.02	0.994 ± 0.02

**Table 5:** Calculation of the thermodynamic descriptors for 2,4-DCP adsorption on different adsorbents.

Adsorbents	T (K)	$lnK_{D}$	${\Delta G}^{0}$ (kJ·mol^−1^)	${\Delta H}^{0}$ (kJ·mol^−1^)	${\Delta S}^{0}$ (kJ·mol^−1^·K^−1^)
PAni	298 ± 0.5	0.48 ± 0.3	−1.19 ± 0.2	−7.15 ± 0.2	−0.02 ± 0.2
	308 ± 0.5	0.38 ± 0.2	−0.99 ± 0.2		
	318 ± 0.3	0.29 ± 0.2	−0.79 ± 0.2		
	328 ± 0.4	0.21 ± 0.5	−0.59 ± 0.2		
PAni:PPy	298 ± 0.5	1.27 ± 0.5	−3.14 ± 0.2	−12.08 ± 0.2	−0.03 ± 0.2
	308 ± 0.4	1.11 ± 0.4	−2.84 ± 0.2		
	318 ± 0.5	0.96 ± 0.4	−2.54 ± 0.2		
	328 ± 0.4	0.82 ± 0.4	−2.24 ± 0.2		
PAni:PPy@SiO_2_	298 ± 0.4	4.57 ± 0.3	−11.32 ± 0.2	−94.76 ± 0.2	−0.28 ± 0.2
	308 ± 0.5	3.33 ± 0.5	−8.52 ± 0.2		
	318 ± 0.3	2.16 ± 0.3	−5.72 ± 0.2		
	328 ± 0.5	1.07 ± 0.3	−2.92 ± 0.2		

## Data Availability

Not applicable.

## References

[1] Mao W., Zhang Y., Luo J., Chen L., Guan Y. (2022). Novel co-polymerization of polypyrrole/polyaniline on ferrate modified biochar composites for the efficient adsorption of hexavalent chromium in water. Chemosphere.

[2] Khan A., Bhatti H.N., Tahira M., Alqahtani F.O., Al-Fawzan F.F., Alissa S.A., Iqbal M. (2023). Na-alginate, polyaniline and polypyrrole composites with cellulosic biomass for the adsorptive removal of herbicide: Kinetics, equilibrium and thermodynamic studies. Arab. J. Chem..

[3] Bhatti H.N., Mahmood Z., Kausar A., Yakout S.M., Shair O.H., Iqbal M. (2020). Biocomposites of polypyrrole, polyaniline and sodium alginate with cellulosic biomass: Adsorption-desorption, kinetics and thermodynamic studies for the removal of 2,4-dichlorophenol. Int. J. Biol. Macromol..

[4] Weng X., Ma H., Owens G., Chen Z. (2023). Enhanced removal of 2,4-dichlorophenol by Fe-Pd@ZIF-8 via adsorption and dechlorination. Sep. Purif. Technol..

[5] Elkady M.F., Hassan H.S. (2021). Photocatalytic Degradation of Malachite Green Dye from Aqueous Solution Using Environmentally Compatible Ag/ZnO Polymeric Nanofibers. Polymers.

[6] Tang Y., He D., Guo Y., Qu W., Shang J., Zhou L., Pan R., Dong W. (2020). Electrochemical oxidative degradation of X-6G dye by boron-doped diamond anodes: Effect of operating parameters. Chemosphere.

[7] Nawaz H., Umar M., Nawaz I., Ullah A., Khawar M.T., Nikiel M., Razzaq H., Siddiq M., Liu X. (2022). Hybrid PVDF/PANI Membrane for Removal of Dyes from Textile Wastewater. Adv. Energy Mater..

[8] Jorge N., Teixeira A.R., Marchão L., Tavares P.B., Lucas M.S., Peres J.A. (2022). Removal of Methylene Blue from Aqueous Solution by Application of Plant-Based Coagulants. Eng. Proc..

[9] Benchikh I., Dahou F.Z., Lahreche S., Sabantina L., Benmimoun Y., Benyoucef A. (2022). Development and characterisation of novel hybrid materials of modified ZnO-SiO_2_ and polyaniline for adsorption of organic dyes. Int. J. Environ. Anal. Chem..

[10] Srikhaow A., Chaengsawang W., Kiatsiriroat T., Kajitvichyanukul P., Smith S.M. (2022). Adsorption Kinetics of Imidacloprid, Acetamiprid and Methomyl Pesticides in Aqueous Solution onto Eucalyptus Woodchip Derived Biochar. Minerals.

[11] Lv H.W., Jiang H.L., He F.A., Hu Q.D., Zhong Z.R., Yang Y.Y. (2022). Adsorption of anionic and cationic dyes by a novel crosslinked cellulose-tetrafluoroterephthalonitrile-tannin polymer. Eur. Polym. J..

[12] German N., Ramanaviciene A., Ramanavicius A. (2019). Formation of Polyaniline and Polypyrrole Nanocomposites with Embedded Glucose Oxidase and Gold Nanoparticles. Polymers.

[13] Gaikwad P.D., Shirale D.J., Gade V.K., Savale P.A., Kakde K.P., Kharat H.J., Shirsat M.D. (2006). Potentiometric study of polyaniline film synthesized with various dopants and composite-dopant: A comparative study. Bull. Mater. Sci..

[14] Luo S.C. (2013). Conducting polymers as biointerfaces and biomaterials: A perspective for a special issue of polymer reviews. Polym. Rev..

[15] Sharma S., Sudhakara P., Omran A.A.B., Singh J., Ilyas A. (2021). Recent Trends and Developments in Conducting Polymer Nanocomposites for Multifunctional Applications. Polymers.

[16] Stejskal J. (2022). Recent Advances in the Removal of Organic Dyes from Aqueous Media with Conducting Polymers, Polyaniline and Polypyrrole, and Their Composites. Polymers.

[17] Belhadj H., Moulefera I., Sabantina L., Benyoucef A. (2022). Effects of Incorporating Titanium Dioxide with Titanium Carbide on Hybrid Materials Reinforced with Polyaniline: Synthesis, Characterization, Electrochemical and Supercapacitive Properties. Fibers.

[18] Boutaleb N., Dahou F.Z., Djelad H., Sabantina L., Moulefera I., Benyoucef A. (2022). Facile Synthesis and Electrochemical Characterization of Polyaniline@TiO_2_-CuO Ternary Composite as Electrodes for Supercapacitor Applications. Polymers.

[19] Lahreche S., Moulefera I., El Kebir A., Sabantina L., Kaid M., Benyoucef A. (2022). Application of Activated Carbon Adsorbents Prepared from Prickly Pear Fruit Seeds and a Conductive Polymer Matrix to Remove Congo Red from Aqueous Solutions. Fibers.

[20] Zhang W., Ou J., Tang M., He Q., Long A., Luo S., Sun S., Wan J., Gao Y., Zhou L. (2022). Physically-crosslinked activated CaCO_3_/polyaniline-polypyrrole-modified GO/alginate hydrogel sorbent with highly efficient removal of copper(II) from aqueous solution. Chem. Eng. J..

[21] Langmuir I. (1916). The constitution and fundamental properties of solids and liquids. Part I. Solids. J. Am. Chem. Soc..

[22] Liu Y., Liu Y.-J. (2008). Biosorption isotherms, kinetics and thermodynamics. Sep. Purif. Technol..

[23] Liu Y., Xu H. (2007). Equilibrium, thermodynamics and mechanisms of Ni^2+^ biosorption by aerobic granules. Biochem. Eng. J..

[24] Liu H., Ji S., Yang H., Zhang H., Tang M. (2014). Ultrasonic-assisted ultra-rapid synthesis of monodisperse meso-SiO_2_@Fe_3_O_4_ microspheres with enhanced mesoporous structure. Ultrason. Sonochem..

[25] Zhou J., Huang W., Qiu B., Hu Q., Cheng X., Guo Z. (2022). Core-shell structured polyaniline/polypyrrole composites promoted methane production from anaerobic sludge. Chemosphere.

[26] Marcelo A.M., Simoes L.G.P., Tremiliosi G.C., Coelho D., Minozzi D.T., Santos R.I., Vilela D.C.B., do Santos J.R., Ribeiro L.K., Rosa I.L.V. (2021). SiO_2_-Ag Composite as a Highly Virucidal Material: A Roadmap that Rapidly Eliminates SARS-CoV-2. Nanomaterials.

[27] Hlekelele L., Nomadolo N.E., Setshedi K.Z., Mofokeng L.E., Avashnee Chetty A., Chauke V.P. (2019). Synthesis and characterization of polyaniline, polypyrrole and zero-valent iron-based materials for the adsorptive and oxidative removal of bisphenol-A from aqueous solution. RSC Adv..

[28] Toumi I., Djelad H., Chouli F., Benyoucef A. (2022). Synthesis of PANI@ZnO Hybrid Material and Evaluations in Adsorption of Congo Red and Methylene Blue Dyes: Structural Characterization and Adsorption Performance. J. Inorg. Organomet. Polym. Mater..

[29] Gupta S.P., Nishad H.H., Chakane S.D., Gosavi S.W., Late D.J., Walke P.S. (2020). Phase transformation in tungsten oxide nanoplates as a function of post-annealing temperature and its electrochemical influence on energy storage. Nanoscale Adv..

[30] Gusev G.I., Gushchin A.A., Grinevich V.A., Fillipov D.V., Moskalenko E.A., Shil’ke M.A. (2021). Adsorption of 2,4-Dichlorophenol and Phenol from Aqueous Solutions by Silicate Sorbent. Russ. J. Phys. Chem..

[31] Mahjoubi F.Z., Khalidi A., Abdennouri M., Barka N. (2016). M-Al-SO_4_ layered double hydroxides (M=Zn, Mg or Ni): Synthesis, characterization and textile dyes removal efficiency. Desalin. Water Treat..

[32] Raoov M., Sharifah Mohamad S., Abas M.R. (2013). Removal of 2,4-dichlorophenol using cyclodextrin-ionic liquid polymer as a macroporous material: Characterization, adsorption isotherm, kinetic study, thermodynamics. J. Hazard. Mater..

[33] Liu Q.S., Zheng T., Wang P., Jiang J.P., Li N. (2010). Adsorption isotherm, kinetic and mechanism studies of some substituted phenols on activated carbon fibers. Chem. Eng. J..

[34] Noreen S., Bhatti H.N., Iqbal M., Hussain F., Sarim F.M. (2020). Chitosan, starch, polyaniline and polypyrrole biocomposite with sugarcane bagasse for the efficient removal of Acid Black dye. Int. J. Biol. Macromol..

[35] Qasem K.M.A., Khan S., Chinnam S., Saleh H.A.M., Mantasha I., Zeeshan M., Mane Y.K., Shahid M. (2022). Sustainable fabrication of Co-MOF@CNT nano-composite for efficient adsorption and removal of organic dyes and selective sensing of Cr(VI) in aqueous phase. Mater. Chem. Phys..

[36] Hajjaoui H., Khnifira M., Soufi A., Abdennouri M., Kaya S., Akkaya R., Barka N. (2022). Experimental, DFT and MD simulation studies of Mordant Black 11 dye adsorption onto polyaniline in aqueous solution. J. Mol. Liq..

[37] Jianming P.J., Zou X., Wang X., Guan W., Li C., Yan Y., Wu X. (2011). Adsorptive removal of 2,4-didichlorophenol and 2,6-didichlorophenol from aqueous solution by cyclodextrin/attapulgite composites: Equilibrium, kinetics and thermodynamics. Chem. Eng. J..

[38] Chai X., Zhao Y. (2006). Adsorption of phenolic compound by aged-refuse. J. Hazard. Mater..

[39] Crini N.M., Winterton P., Fourmentin S., Wilson L.D., Fenyvesi E., Crini G. (2018). Water-insoluble β-cyclodextrin–epichlorohydrin polymers for removal of pollutants from aqueous solutions by sorption processes using batch studies: A review of inclusion mechanisms. Prog. Polym. Sci..

[40] Sathishkumar M., Binupriya A.R., Kavitha D., Yun S.E. (2007). Kinetic and isothermal studies on liquid-phase adsorption of 2,4-dichlorophenol by palm pith carbon. Bioresour. Technol..

[41] Edet U.A., Ifelebuegu A.O. (2020). Kinetics, Isotherms, and Thermodynamic Modeling of the Adsorption of Phosphates from Model Wastewater Using Recycled Brick Waste. Processes.

